# Cellular metabolism and homeostasis in pluripotency regulation

**DOI:** 10.1007/s13238-020-00755-1

**Published:** 2020-07-08

**Authors:** Kun Liu, Jiani Cao, Xingxing Shi, Liang Wang, Tongbiao Zhao

**Affiliations:** 1 State Key Laboratory of Stem Cell and Reproductive Biology, Institute of Zoology, Chinese Academy of Sciences, Beijing, 100101 China; 2 Institute for Stem Cell and Regeneration, Chinese Academy of Sciences, Beijing, 100101 China; 3 University of Chinese Academy of Sciences, Beijing, 100049 China

**Keywords:** autophagy, amino acid metabolism, lipid metabolism, pluripotent stem cell (PSC), ubiquitin-proteasome system (UPS)

## Abstract

Pluripotent stem cells (PSCs) can immortally self-renew in culture with a high proliferation rate, and they possess unique metabolic characteristics that facilitate pluripotency regulation. Here, we review recent progress in understanding the mechanisms that link cellular metabolism and homeostasis to pluripotency regulation, with particular emphasis on pathways involving amino acid metabolism, lipid metabolism, the ubiquitin-proteasome system and autophagy. Metabolism of amino acids and lipids is tightly coupled to epigenetic modification, organelle remodeling and cell signaling pathways for pluripotency regulation. PSCs harness enhanced proteasome and autophagy activity to meet the material and energy requirements for cellular homeostasis. These regulatory events reflect a fine balance between the intrinsic cellular requirements and the extrinsic environment. A more complete understanding of this balance will pave new ways to manipulate PSC fate.

## Introduction

Pluripotent stem cells (PSCs), including embryonic stem cells (ESCs) and induced pluripotent stem cells (iPSCs), have the capacity to self-renew and differentiate into all cell types of our bodies (Liu et al., 2014; Martello and Smith, 2014). These properties depend on a series of pluripotency genes that are highly expressed and coordinately regulated in PSCs (Boyer et al., 2005; Orkin and Hochedlinger, 2011). At the same time, PSCs have developed unique cell cycle characteristics and a high proliferation rate to match the activity of pluripotency gene networks (Wang et al., 2008; Singh and Dalton, 2009). Emerging evidence shows that metabolic pathways are mediators of crosstalk between cellular degradation, cellular recycling, epigenetic regulation, signal transduction and stem cell fate determination (Folmes et al., 2012; Buck et al., 2016; Gascon et al., 2016; Zhang et al., 2016b; Zheng et al., 2016).

Cellular metabolism, including anabolism and catabolism, involves multiple complex biochemical processes, such as amino acid metabolism, nucleic acid metabolism, fatty acid metabolism, glycolysis, oxidative phosphorylation, the ubiquitin-proteasome degradation system, autophagy, and so on (Naujokat and Saric, 2007; Vessoni et al., 2012; Kilberg et al., 2016; Wang et al., 2017). Metabolism is not only involved in energy production and degradation and biosynthesis of cellular components, but also takes part in signal transduction for genetic and epigenetic regulation through its intermediate metabolites (Zhang et al., 2018). Cells utilize anabolism to produce biological macromolecules and organelles. Catabolism is responsible for degrading and recycling both normal and harmful substances and dysfunctional organelles by breaking them down into small molecules (Mathieu and Ruohola-Baker, 2017). Anabolism and catabolism are tightly coordinated and essential for maintaining cell proliferation and function (Zhang et al., 2014; Kaur and Debnath, 2015).

Existing studies have proposed that PSCs mainly rely on glycolysis for energy generation, while somatic cells prefer oxidative phosphorylation for ATP production; this issue has been vigorously discussed by several excellent reviews (Folmes et al., 2012; Zhang et al., 2012; Xu et al., 2013; Mathieu and Ruohola-Baker, 2017; Zhang et al., 2018). Here we mainly summarize recent research progress in understanding how pluripotency is regulated by metabolic pathways involving amino acids, fatty acids, the ubiquitin-proteasome system and autophagy.

## Amino acid metabolism and pluripotency

The important role of amino acids in the later stages of preimplantation embryo development was firstly noted by Gwatkin while he was formulating mouse embryo culture medium. He initially found that adding amino acids into the culture medium induced the attachment and outgrowth of mouse blastocysts (Gwatkin, 1966). Following these observations, Spindle and Pedersen demonstrated that adding amino acids into culture medium not only improved attachment and outgrowth of blastocysts but also increased embryo hatching rate (Spindle and Pedersen, 1973). It was later found that preimplantation embryos have transporters for specific amino acids and can maintain an endogenous pool of amino acids (Schultz et al., 1981; Sellens et al., 1981; Van Winkle, 2001).

In cellular biosynthetic pathways, amino acids are essential nutrients that contribute directly to protein synthesis as well as providing compounds for chemical modifications. Although the amino acid metabolism and protein synthesis pathways are well defined in somatic cells, relatively little is known about the contribution of amino acid metabolism to pluripotency regulation in PSCs. Emerging studies provide evidence that amino acids regulate pluripotency by providing chemical groups for chromatin modifications (Kilberg et al., 2016; D’Aniello et al., 2019).

### Threonine metabolism and pluripotency

**Figure 1:**
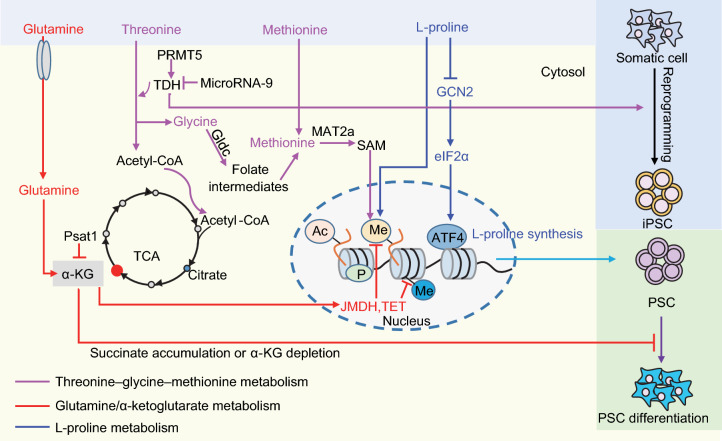
**Amino acid metabolism in pluripotency regulation**. Threonine/methionine metabolism contribute to pluripotency regulation by providing SAM for DNA and histone methylation in PSCs. The threonine dehydrogenase TDH is highly expressed in mESCs, maintaining a high ratio of SAM/SAH that is correlated with high H3K4me3 levels. TDH expression is positively regulated by PRMT5 and negatively regulated by microRNA-9. Metabolism of glutamine and glucose regulates pluripotency through α-KG, which is a cofactor for Jumonji domain-containing histone demethylases (JMDH) and the ten-eleven translocation family of enzymes (TETs) that are involved in DNA demethylation. The cellular level of L-proline is fine-tuned by the amino acid starvation response (AAR) pathway *Gcn2-Eif2α-Atf4*. Excessive supplementation with L-proline leads to ESC differentiation. Appropriate intracellular synthesis of L-proline safeguards PSC pluripotency

Threonine is an essential amino acid, which can be catabolized by threonine dehydrogenase (TDH) to glycine and acetyl-coenzyme A (acetyl-CoA). Glycine can be catabolized by glycine decarboxylase (Gldc) to generate folate intermediates to fuel one-carbon metabolism. The folate intermediates fuel nucleotide synthesis and remethylation of homocysteine to form methionine (Met) and S-adenosyl-methionine (SAM). SAM serves as a universal methyl donor for DNA and histone methylation reactions. Acetyl-CoA feeds the tricarboxylic acid (TCA) cycle as well as serving as the donor of acetyl groups (Fig. 1).

Mouse ESCs (mESCs) exist in a high-flux metabolic state that depends on threonine catabolism. By depriving mESCs of each of the 20 amino acids individually, Wang et al. have shown that threonine catabolism is required for mESC identity maintenance (Wang et al., 2009). The underlying mechanism was revealed by Shyh-Chang et al.: threonine maintains mESC pluripotency by providing SAM for trimethylation of histone H3 lysine 4 (H3K4me3) (Shyh-Chang et al., 2013).

In addition, Alexander et al. have shown that mESCs rapidly discontinue DNA synthesis, arrest cell division and eventually die after threonine deprivation (Alexander et al., 2011). When threonine is withdrawn for 1 to 4 days, the expression of pluripotency marker genes decreases, accompanied by increased expression of trophoectodermal and mesodermal marker genes. Refeeding threonine one day after deprivation leads to increased expression of cyclin D1 and E in mESCs. They proposed that threonine regulates mESC proliferation by stimulating G_1_/S transition through lipid raft/caveolae-dependent PI3K/Akt, MAPKs, mTOR, p70S6K, and 4E-BP1 signaling pathways (Ryu and Han, 2011; Kilberg et al., 2016).

The enzyme L-threnonine-3-dehydrogenase (TDH) is highly expressed in mESCs and is the first and rate-limiting enzyme in the pathway that hydrolyzes threonine into glycine and acetyl-CoA in mitochondria. High activity and expression of TDH maintain a high SAM/SAH(S-adenosylhomocysteine) ratio in mESCs, which is tightly correlated with H3K4me3 levels (Wang et al., 2009; Ang et al., 2011; Shyh-Chang et al., 2013). During reprogramming, induction of TDH enhances, whereas knockdown of TDH inhibits, reprogramming efficiency (Han et al., 2013). TDH expression can be negatively regulated at the post-transcriptional level by microRNA-9, and positively regulated by protein arginine methyltransferase (PRMT5) (Han et al., 2013).

### Methionine metabolism and pluripotency

Methionine metabolism is involved in epigenetic maintenance, redox homeostasis and organism development (Tang et al., 2017). Methionine can be directly converted to SAM in a reaction catalyzed by methionine adenosyltransferase 2a (MAT2a), which provides a threonine-independent route for generating SAM. SIRT1, the most conserved mammalian NAD^+^-dependent protein deacetylase, critically regulates the activity of MAT2a in coordination with *Myc*. Deletion of SIRT1 in mESCs increased the hypersensitivity of the cells to methionine restriction/depletion-induced differentiation and apoptosis (Tang et al., 2017).

Unlike the situation in mESCs, absence of threonine does not evidently affect the pluripotency of human ESCs (hESCs), as the TDH gene is a non-functional pseudogene in humans (Edgar, 2002). Instead, methionine directly produces SAM in hESCs. Depletion of methionine induces a rapid decrease of intracellular SAM in hPSCs, resulting in decreased global H3K4me3, activation of p53-p38 signaling, reduction of NANOG expression, and thereafter differentiation and apoptosis of hPSCs (Shiraki et al., 2014). Interestingly, the precise level of SAM, which is restricted by a nicotinamide N-methyltransferase (NNMT)-dependent SAM-consuming pathway, is critical for maintaining the naïve state of hESCs. NNMT consumes SAM to keep it at a low level to preserve the naïve state of hESCs, while a high level of SAM promotes the naïve-to-primed transition through H3K27me3 (Sperber et al., 2015).

### L-proline (L-Pro) and pluripotency

The intracellular concentration of L-Pro in mESCs is around 4-fold lower than in mESCs in the L-Pro-induced primed state, and 10-fold lower than in mouse embryonic fibroblasts. The cellular level of L-Pro in mESCs is strictly regulated by the *Gcn2-Eif2α-Atf4* amino acid starvation response (AAR) pathway (D’Aniello et al., 2015). The low level of intracellular L-Pro induces expression of *Atf4* to enhance intracellular synthesis of L-Pro through activation of *Aldh18a1*/*Pycr1*; this maintains a naïve state of pluripotency that is less dependent on exogenous L-Pro supplementation. Excessive supplementation with L-Pro inactivates *Gcn2-Eif2α-Atf4* AAR and thus inhibits intracellular L-Pro synthesis, resulting in mESC mesenchymal transition. This feedback regulatory loop precisely maintains the appropriate intrinsic L-Pro level, which restricts proliferation of tightly packed dome-like mESC colonies and safeguards mESC identity (Fig. 1).

Differentiation of pluripotent cells within the mammalian blastocyst starts with the formation of the primitive ectoderm, or epiblast, from the inner cell mass (ICM). This early differentiation step can be recaptured *in vitro* by culturing mESCs in MEDII conditioned medium, which leads to formation of primitive ectoderm-like cells. Supplementation with L-Pro promotes, whereas depletion of L-Pro uptake inhibits, the differentiation of mESCs toward primitive ectoderm (Washington et al., 2010; Kilberg et al., 2016). In support of this observation, another independent study identified that L-Pro induces mESCs toward an epiblast-like stem cell (EpiSCs) phenotype in a dose- and time- dependent manner. This EpiSC phenotype induced by L-Pro can be reversed by either withdrawal of L-Pro or addition of L-ascorbic acid (vitamin C, Vc) (Casalino et al., 2011).

Following on from these studies, Comes et al. showed that the phenotype remodeling in EpiSCs is reminiscent of a change toward a mesenchymal-like state. They proposed that L-Pro serves as a signaling molecule to promote mESC differentiation by stimulation of the epithelial-to-mesenchymal transition (Comes et al., 2013). Interestingly, this ESC-to-mesenchymal transition is accompanied by a genome-wide increase of DNA methylation and specific histone modifications (H3K9me2/H3K9me3 and H3K36me3) (Comes et al., 2013; D’Aniello et al., 2017). These changes in histone modification can be reversed by either removal of L-Pro or treatment with Vc. Conversely, somatic cell reprogramming to iPSCs involves a mesenchymal-to-epithelial transition, which can be promoted by Vc through the H3K36 demethylase Jhdm1a/1b (Wang et al., 2011). Thus, L-Pro antagonizes Vc in terms of DNA methylation and chromatin structure, which leads to opposing effects on pluripotency regulation.

### Glutamine/α-Ketoglutarate and pluripotency

In mESCs, glutamine can be catabolized to α-ketoglutarate (α-KG) to support TCA cycle anaplerosis. α-KG is an important metabolic intermediate that acts as a cofactor for Jumonji domain-containing histone demethylases (JMDH) and the ten-eleven translocation family of enzymes (TETs) that are involved in DNA demethylation (Fig. 1). Glutamine contributes to pluripotency by generating α-KG to regulate cellular DNA/histone methylation states.

Naïve mESCs use both glucose and glutamine catabolism to generate a high level of cellular α-KG, which contributes to a low level of intracellular H3K27me3 and increases TET-dependent DNA demethylation (Carey et al., 2015). Direct manipulation of the intracellular α-KG level is sufficient to change the levels of cellular H3K27me3 and TET-dependent DNA demethylation, which are correlated with pluripotency gene expression. Cellular α-KG levels can be regulated by phosphoserine aminotransferase1 (Psat1), which is an *Oct4*/*Sox2*/*Nanog* target protein. Decreased expression of *Psat1* in mESCs lowers the levels of DNA 5’-hydroxymethylcytosine and H3K9me3, resulting in accelerated differentiation of mESCs (Hwang et al., 2016).

## Fatty acid synthesis and pluripotency

**Figure 2:**
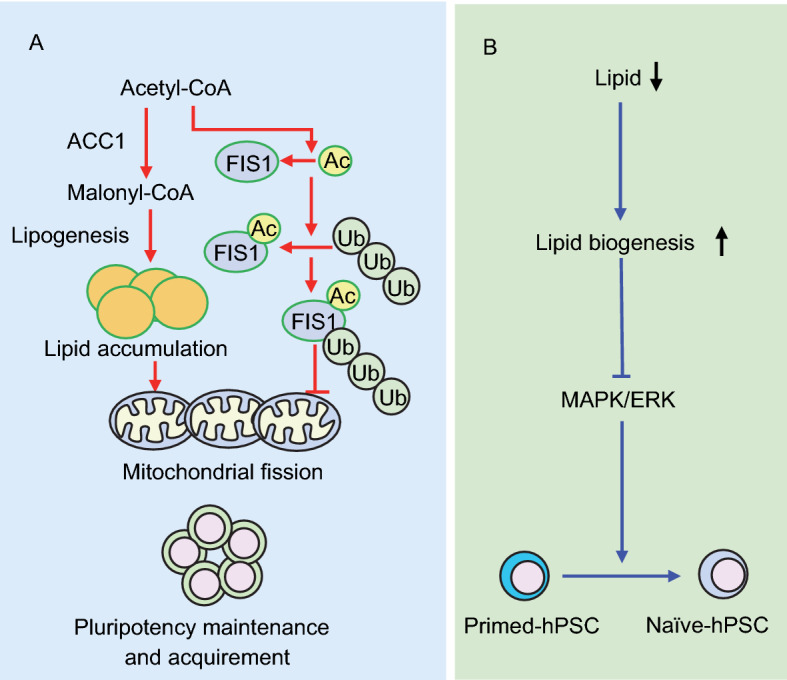
**Fatty acid metabolism in pluripotency regulation**. Appropriate cellular levels of lipids safeguard pluripotency. (A) *De novo* synthesis of fatty acids, initiated by the enzyme acetyl-Coenzyme A carboxylase alpha (ACC1), promotes pluripotency maintenance and acquisition by enhancing mitochondrial fission. This conserved pathway is antagonized by ubiquitin-proteasome mediated degradation of the acetylated mitochondrial fission mediator FIS1. (B) In human PSCs, exogenous lipid deficiency induces intracellular lipogenesis which ultimately inhibits endogenous ERK and promotes pluripotency

Lipids serve as the predominant components of plasma and organelle membranes, as secondary messengers for signal transduction, and as an important source of energy. In a search for new molecules that contribute to long-term hESC self-renewal, Garcia-Gonzalo and Izpisua Belmonte identified that the lipids in lipid-rich albumin from a chemically-defined medium stimulated hESC pluripotency (Garcia-Gonzalo and Izpisua Belmonte, 2008). This provided the first evidence to show that lipids contribute to pluripotency regulation. Using mass spectrometry-based metabolomics analysis, Yanes et al. identified that unsaturated lipid levels decreased upon mESC differentiation (Yanes et al., 2010). Inhibition of the eicosanoid signaling pathway maintained the levels of unsaturated fatty acids and improved pluripotency. In accordance with these observation, Wang et al. demonstrated that *de novo* synthesis of fatty acids, which is mediated by the rate-limiting enzyme ACC1 (acetyl-Coenzyme A carboxylase alpha), is required for pluripotency acquisition and maintenance (Wang et al., 2017). Activation of ACC1 leads to decreased acetyl-CoA production and increased cellular lipid generation, which promotes mitochondrial fission and cellular pluripotency (Fig. 2A). This pathway is conserved in both human and mouse ESCs (Wang et al., 2017). Conversely, Dunning et al. showed that promoting fatty acid beta-oxidation by carnitine supplementation during *in vitro* culture of early mouse embryos increased the blastocyst hatching rate and the ICM cell number (Dunning et al., 2010). The reason for this discrepancy is unclear. One possible interpretation is that the balance between fatty acid oxidation and fatty acid synthesis maintains an appropriate cellular fatty acid level in ESCs that facilitates pluripotency regulation.

By comprehensive metabolic flux analysis of hESCs cultured either in mouse embryonic fibroblast (MEF)-conditioned medium or essential 8 minimal medium (E8, no lipids), Zhang et al. demonstrated that the lack of lipids in E8 medium promotes oxidative pentose phosphate pathway (PPP) flux for NADPH synthesis, *de novo* lipogenesis and reductive carboxylation, while simultaneously decreasing mitochondrial respiration. Supplementation of E8 medium with lipids resulted in decreased lipogenesis and increased oxidative mitochondrial metabolism in hESCs (Zhang et al., 2016a). Most recently, Cornacchia et al. demonstrated that hESCs cultured in E8 medium captured a naïve-to-primed intermediate state of pluripotency with *de novo* lipogenesis and endogenous ERK inhibition that mimics *in vivo* regulation during pre-implantation development (Fig. 2B) (Cornacchia et al., 2019).

Together, these data indicate that *de novo* lipogenesis and appropriate cellular levels of lipids play critical roles in pluripotency regulation in both mouse and human PSCs. In addition, these studies deliver supporting evidence that mitochondrial respiration contributes to pluripotency maintenance, which challenges the traditional notion that mitochondrial function is dispensable for pluripotent stem cell function.

## The ubiquitin-proteasome system and pluripotency

**Figure 3:**
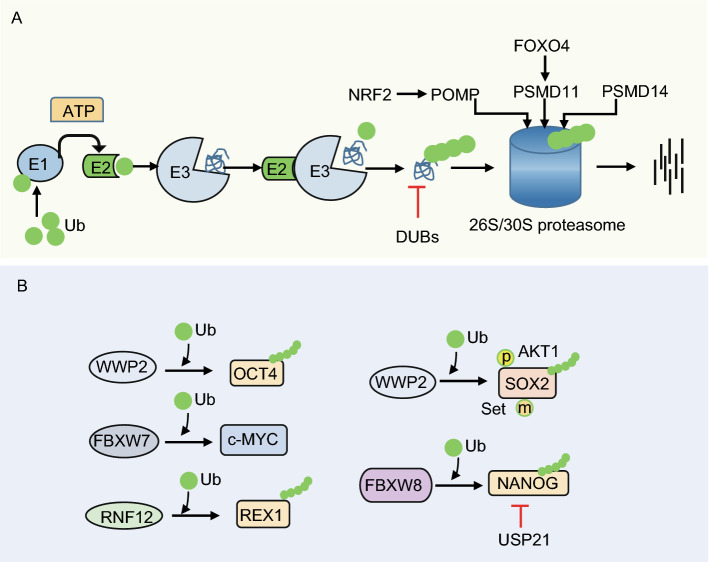
**Regulation of pluripotency by the ubiquitin-proteasome system (UPS)**. (A) PSCs exhibit high proteasome degradation activity, which is regulated by FOXO4-driven expression of the 19S proteasome subunit PSMD11 and corresponding enhanced assembly of 26S/30S proteasomes. (B) Levels of the pluripotency factors OCT4, c-MYC, REX1, SOX2 and NANOG are fine-tuned by UPS to maintain the precise quantity that facilitates pluripotency. p, phosphorylation; m, methylation

Ubiquitination is a cascade enzymatic reaction that involves covalent addition of ubiquitin (Ub) to target proteins (Fig. 3A). The E1 Ub-activating enzyme activates Ub in an ATP-dependent manner, and forms a thioester bond between a cysteine in the enzyme and the carboxyl terminus of Ub. Then, activated Ub is transferred to E2 (Ub carrier enzyme or Ub-conjugating enzyme). Delivery of the Ub by E2 to the target protein is dependent on the E3 Ub protein ligase. The E3 enzyme is responsible for substrate recognition and for promoting the elongation of Ub chains (Weissman, 2001). Deubiquitinating enzymes (DUBs) are required to specifically disassemble ubiquitin chains, thus balancing ubiquitination and deubiquitination (Chandrasekaran et al., 2017).

The 26S/30S proteasome consists of the 20S core structure (which contains the proteolytic active site) and 19S cap structures (which regulate the activity of the holo-complex 26S, single and 30S, double capped) (Finley, 2009). The proteasome has caspase-, trypsin-, and chymotrypsin-like activity that degrades ubiquitin-labeled target proteins into 2–24 amino acid peptides (Bedford et al., 2010; Dikic, 2017).

The ubiquitin-proteasome system (UPS) functions in a variety of cellular processes including proliferation, differentiation, apoptosis and senescence (Mayer, 2000). The cardinal role of ubiquitination is to generate poly-ubiquitinated proteins that are recognized and degraded by the 26S proteasome (Weissman, 2001).

Early studies have shown that ubiquitin ligases are involved in degradation of pluripotency factors (Fig. 3B). For example, Xu et al. demonstrated that WWP2, an E3 ubiquitin (Ub)-protein ligase, interacts with the pluripotency protein OCT4 and enhances its Ub modification in both mouse and human ESCs (Xu et al., 2004; Xu et al., 2009). Accordingly, disruption of WWP2-mediated OCT4 ubiquitination promotes reprogramming of mouse embryonic fibroblasts (Li et al., 2018). Interestingly, WWP2 specifically interacts with Set7-methylated SOX2 and induces SOX2 ubiquitination and degradation in mESCs, which can be antagonized by AKT1-mediated phosphorylation at T118. AKT1 activity dominates over Set7 to maintain SOX2 stability and pluripotency in mESCs (Fang et al., 2014). In addition, the X-encoded E3 ubiquitin ligase RNF12 has been identified to mediate degradation of REX1 in a dose-dependent manner in mESCs, thereby contributing to X-chromosome inactivation (Jonkers et al., 2009; Gontan et al., 2012). Furthermore, Ddb1, a component of the CUL4-DDB1 E3 ligase complex, was demonstrated to contribute to pluripotency regulation. Deletion of DDB1 results in mESC differentiation (Gao et al., 2015).

In addition to E3 ligases, the E2 ligase UBE2S (ubiquitin-conjugating enzyme E2S) directly interacts with SOX2 at the K123 residue to maintain a proper SOX2 protein level through K11-linked polyubiquitination, thus reinforcing the undifferentiated state of mESCs (Wang et al., 2016).

A high-throughput functional genetic screening study using a cDNA-based random RNA interference library in mESCs revealed that knockdown of ubiquitin increased the formation of ESC colonies in the absence of LIF (Jian et al., 2007). A more comprehensive study using UPS-targeted RNAi screens identified that the DUBs PSMD14 and USP9X; the E3 ligases RBX1, RFWD2, RNF12, UBR5, and DDB1; and the putative E3 ligases TRIM28 and PHF5a contribute to ESC self-renewal, whereas the E3 ligases FBXW7, RNF152, RNF31, RNF8, SOCS3, and TOPORS; the putative ligases RNF36 and TNFRSF25; and the ubiquitin-like protein UBL5 regulate early ESC differentiation (Buckley et al., 2012). The authors provided evidence to show that, mechanistically, the E3 ligase FBXW7 acts as a key regulator of ESC differentiation through regulation of c-MYC stability. Silencing of *Fbxw7* expression inhibited ESC differentiation and enhanced cellular reprogramming through stabilization of c-MYC. In contrast, the DUB PSMD14 maintains mESC self-renewal by deubiquitination and subsequent degradation of target proteins. Knockdown of *Psmd14* significantly inhibits mESC self-renewal and pluripotency (Buckley et al., 2012). In support of these findings, subsequent studies have shown that the DUB USP21 maintains mESC stemness via stabilization of NANOG (Jin et al., 2016; Liu et al., 2016b; Kwon et al., 2017). A motif rich in proline, glutamine, serine, and threonine from amino acids 47 to 72 in the N-terminus of NANOG was identified as the target for degradation (Ramakrishna et al., 2011). In addition, the prolyl isomerase PIN1 interacts with and stabilizes NANOG by inhibiting NANOG ubiquitination, thus maintaining pluripotency in both mouse and human ESCs (Moretto-Zita et al., 2010).

In parallel with these findings, Szutorisz et al. have demonstrated that proteasomes target transcription factors and RNA polymerase II bound at tissue-specific gene domains in mESCs to restrict permissive transcription and maintain these genes in a poised state, facilitating the following differentiation initiations (Szutorisz et al., 2006).

Interestingly, Vilchez et al. have shown that hESCs exhibit high proteasome activity, which is characterized by increased levels of the 19S proteasome subunit PSMD11 and corresponding enhanced assembly of 26S/30S proteasomes. The expression of PSMD11 is regulated by the insulin/insulin-like growth factor-I (IGF-I)-responsive transcription factor FOXO4 (Vilchez et al., 2012).

Together, these existing studies provide supporting evidence that enhanced proteasome activity in ESCs is essential for cellular proteostasis regulation and pluripotency maintenance. On one hand, components of the UPS interact with multiple pluripotency factors like OCT4, SOX2, NANOG, c-MYC, and REX1 to fine-tune their cellular levels to meet pluripotency requirements; on the other hand, the UPS regulates the degradation of transcription factors and RNA polymerase II bound to tissue-specific genes in ESCs to maintain the pluripotent state of ESCs.

## Autophagic degradation and pluripotency

**Figure 4:**
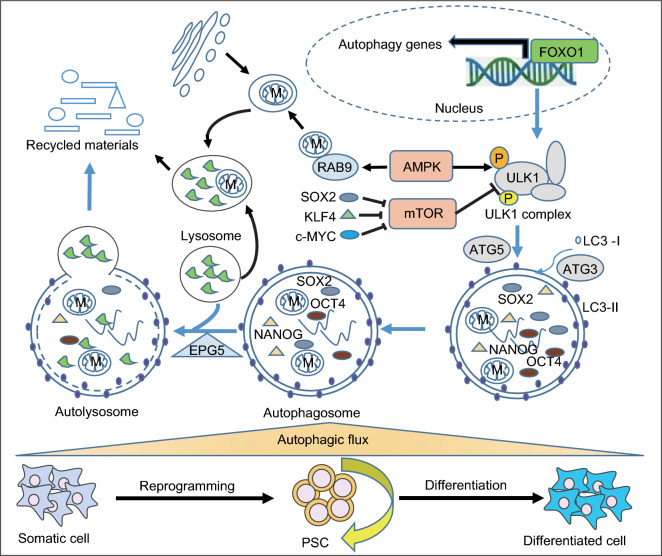
**Regulation of pluripotency by autophagy**. PSCs exhibit a high autophagic flux that is regulated by FOXO1, which coordinates the autophagy machinery gene program at the transcriptional level. High autophagic flux maintains appropriate levels of cellular pluripotency factors like OCT4, SOX2 and NANOG, and organelles like mitochondria (M). Inhibition of autophagy leads to accumulation of abnormal mitochondria and breakdown of pluripotency in spite of increased levels of pluripotency proteins. Activation of autophagy by AMPK is essential for both pluripotency maintenance and acquisition. Inactivation of mTOR by the pluripotency factors SOX2, KLK4 or c-MYC facilitates somatic cell reprogramming to pluripotency

Autophagy is a lysosome-dependent catabolic process, in which cytosolic materials (including proteins, organelles, and lipids) are sequestrated into double-membrane vesicles (termed autophagosome) and delivered to lysosomes for degradation (Klionsky and Emr, 2000). Autophagy recycles damaged or superfluous cytoplasmic contents to maintain cellular homeostasis. Increasing numbers of studies suggest that autophagy plays important roles in regulating ESC cellular homeostasis to maintain self-renewal and pluripotency (Fig. 4).

Autophagy was first revealed to regulate cellular homeostasis in ESCs by Yoshimori’s group, who used *Atg5*-null mESCs to study autophagy mechanisms. They found that ATG5 is critical for autophagosome formation, and ATG5-dependent autophagy accounts for the majority of protein degradation by lysosomes in mESCs (Mizushima et al., 2001). Autophagic flux is maintained by a core molecular machinery which is composed of autophagy sensor systems, the induction complex, and autophagosome formation systems. mESCs possess a powerful autophagic infrastructure with enhanced expression of most of the core autophagy machinery genes compared to somatic fibroblasts (Liu et al., 2017). This substantial infrastructure, regulated by the master transcriptional factor FOXO1, endows mESCs with a high autophagic flux. Downregulation of autophagic flux compromised mESC self-renewal and pluripotency (Liu et al., 2017). Furthermore, deletion of the autophagy regulator *Atg3* in mESCs resulted in increased accumulation of abnormal mitochondria and enhanced production of reactive oxygen species (ROS), together with decreased mitochondrial potential and ATP generation (Liu et al., 2016a).

In the search for molecules that protect pluripotency in mouse ESCs, Gu et al. identified EPG5, a eukaryotic-specific autophagy regulator which mediates autophagosome/lysosome fusion, as a guardian of ESC stemness. Deubiquitination of EPG5 by USP8 at Lys 252 consolidates the interaction between EPG5 and LC3 and thus sustains a high autophagic flux for stemness maintenance (Gu et al., 2019). In addition, the mammalian autophagy-initiating kinase ULK1 is highly expressed in mESCs, and constitutive activation of ULK1 by AMPK (AMP activated protein kinase) functions as an intrinsic signaling pathway in ESCs to regulate their identity under normal physiological conditions (Gong et al., 2018).

An appropriate cellular level of pluripotency proteins in ESCs is critical for pluripotency maintenance. The stability of the pluripotency proteins OCT4, SOX2 and NANOG in hESCs is regulated by autophagy in addition to regulation by the UPS as described above. Autophagy inhibition led to reduction of pluripotency despite accumulation of these pluripotency proteins (Cho et al., 2014).

PSCs are distinct from somatic cells in having a smaller volume and fewer mitochondria. During somatic cell reprogramming, treatment with mTOR (mammalian target of rapamycin) inhibitors like rapamycin or PP242, or the autophagy inducer spermidine, significantly improved the speed and efficiency of iPSC generation, which indicates that autophagy is involved in this cellular remodeling (Chen et al., 2011; Menendez et al., 2011). In support of this proposition, the reprogramming factor SOX2 was identified to directly bind to the promoter of mTOR to recruit the NuRD complex. This resulted in transcriptional repression of mTOR, thus inducing a transient activation of autophagy, which accounted for successful reprogramming (Wang et al., 2013). In addition, KLF4 and c-MYC were found to inhibit mTORC1 (mechanistic target of rapamycin complex 1) during pluripotency acquisition, leading to autophagy activation, mitochondrial remodeling and cell size reduction, which facilitated reprogramming (Wu et al., 2015). Consistent with this, silencing of canonical autophagy by *Atg3* deletion inhibited mitochondrial remodeling during pluripotency induction, resulting in decreased reprogramming efficiency and more abnormal mitochondria in established mouse iPSCs (Liu et al., 2016a). Interestingly, in parallel with these findings, Ma et al. demonstrated that an AMPK-activated *Atg5*-independent autophagy pathway contributes to mitochondrial clearance, and facilitates the metabolic switch from mitochondrial oxidative phosphorylation to glycolysis during somatic cell reprogramming (Ma et al., 2015). Mitophagy, the specific clearance of mitochondria by autophagy, can be initiated by BNIP3L (BCL2/adenovirus E1B interacting protein 3-like). BNIP3L-mediated mitophagy contributes to mitochondrial clearance during reprogramming induced by OCT4/SO2/KLF4 but not OCT4/SO2/KLF4/c-MYC (Xiang et al., 2017).

Taken together, the existing studies deliver supporting evidence that a high level of autophagy activity is required in ESCs to maintain cellular homeostasis of proteins and mitochondria, thus safeguarding pluripotency. During somatic cell reprogramming, both canonical and non-canonical autophagy contribute to cellular remodeling (Fig. 4).

## Future directions

PSCs can potentially provide unlimited resources to benefit regenerative medicine through *in vitro*-directed differentiation. Significant achievements have been made in understanding the mechanistic roles of transcription factors, epigenetic factors and signaling pathways in pluripotency regulation, which will facilitate the optimization of differentiation protocols. However, efficient generation of the desired specific cell type from PSCs remains a major challenge. This is partly because there are still gaps in our knowledge about pluripotency regulation. The recent findings described here—that cellular catabolism, anabolism and homeostasis pathways contribute to pluripotency maintenance—provide new insights into PSC fate determination and pave new ways for stem cell fate manipulation. These findings raise a number of questions: (1) What are the upstream signals for metabolic switching during cell fate transition? (2) How is the balance between anabolism and catabolism regulated in pluripotency acquisition and maintenance? (3) What is the role and mechanism of anabolism and catabolism in regulating reprogramming and differentiation? (4) How does the UPS cooperate with autophagy to regulate pluripotency? (5) How do intermediate metabolites play roles in cell fate transition? (6) Is it possible to optimize PSC differentiation protocols by manipulating anabolism, catabolism, UPS or autophagy? Given the rapid development of omics technologies like proteomics, transcriptomics, epigenomics and metabolomics, together with bioinformatics and artificial intelligence, our understanding of the metabolic regulation of pluripotency will rapidly move forward and thus significantly benefit regenerative medicine.
